# Falls in Progressive Supranuclear Palsy

**DOI:** 10.1002/mdc3.12879

**Published:** 2019-12-19

**Authors:** Fraser S. Brown, James B. Rowe, Luca Passamonti, Timothy Rittman

**Affiliations:** ^1^ Department of Clinical Neurosciences Cambridge University Hospitals Cambridge United Kingdom; ^2^ Department of Clinical Neurosciences University of Cambridge Cambridge United Kingdom; ^3^ Department of Neurology Addenbrooke's Hospital Cambridge United Kingdom

**Keywords:** Falls, progressive supranuclear palsy

## Abstract

**Background:**

Despite falls being an almost universal clinical feature and central to the presentation and diagnostic criteria of progressive supranuclear palsy, our understanding of falls is surprisingly limited and there are few effective treatment options.

**Objectives:**

To provide an overview of the topic of the impact, assessment, mechanism, and management of falls in progressive supranuclear palsy.

**Methods:**

We performed a literature search for “falls” and “progressive supranuclear palsy” and included additional relevant literature known to us. We synthesized this literature with experience from clinical practice.

**Results:**

We review current understanding of the pathophysiology of falls, highlighting the roles of the indirect pathway and the pedunculopontine nucleus. We go on to identify shortcomings in commonly used assessments to measure falls. We discuss medical and nonmedical fall prevention strategies, and finally we discuss balancing falls risk against promoting independence.

**Conclusion:**

Falls are central to progressive supranuclear palsy presentation and diagnosis. Indirect locomotor and pedunculopontine nucleus dysfunction are thought to be the neural substrate of falls in this condition. Attempts to measure and prevent falls, by medical and nonmedical means, are currently limited. A personalized approach is advocated in the management of falls.

Progressive supranuclear palsy (PSP) is a progressive neurodegenerative disease classically presenting with Richardson's syndrome (RS), which is characterized by falls, vertical supranuclear gaze palsy, axial rigidity, and a dysexecutive syndrome with peak onset at age 63.^1^, ^2^ It was first described as a distinct clinical entity in 1964.^3^ Since then there have been several major revisions in its diagnostic criteria and nosology. Early falls feature in the diagnostic criteria for PSP‐RS and constitute a defining clinical feature.^4^, ^5^ The falls of PSP‐RS are unexplained by environmental hazards, loss of consciousness, or cardiovascular causes and are disproportionately common backward.^4^

Despite the fact that falls are almost a universal clinical feature and central to the diagnosis, our understanding of falls in PSP is surprisingly limited, and there are few effective treatment options. In older adults, injuries as a result of falls are a leading cause of death and hospital admission and are associated with significant medical costs.^6^, ^7^, ^8^ In people with PSP, 28.6% develop a fracture from falling, compared with 19.8% in people with other forms of atypical parkinsonism (eg, multiple system atrophy and corticobasal syndromes), and an approximately 5% to 15% 10‐year probability of fracture in the general population aged older than 75.^9^, ^10^

Data are lacking on the prevalence and impact of falls in people with pathologically confirmed PSP and with phenotypes other than PSP‐RS. For example, some people with PSP pathology can initially present with clinical features overlapping with idiopathic Parkinson's disease.^4^ Other presentations include a behavioral syndrome similar to behavioral variant frontotempoaral dementia, corticobasal syndrome, and progressive gait freezing. Although people with PSP may present with these alternative syndromes, most go on to develop key features of RS within 5 years, including falls.^11^

In this review, we concentrate on the PSP‐RS syndrome where falls occur early and are central to the clinical presentation. We review why people with PSP fall and the neuroanatomy and mechanisms underlying this phenomenon. We then examine the natural history of falls and consider how best to measure falls risk and balance. We consider the potential strategies to prevent falls in PSP and reduce their harmful consequences. Finally, we weigh the risks versus benefits of continuing to mobilize and advocate for a positive risk‐taking approach.

## Methods

The PUBMED database was searched with the terms progressive supranuclear palsy AND fall, progressive supranuclear palsy AND recurrent falls, progressive supranuclear palsy AND fall risk, progressive supranuclear palsy AND multiple falls. Records yielded from the search were checked for comparisons between groups of frequent versus infrequent fallers to attempt to identify factors associate with falls. We included additional relevant published literature known to us. Limited evidence was identified, and as such we performed a structured review of the topic and synthesized this with experience from clinical practice.

## Results

### The Neuroanatomy and Mechanism of Falls

PSP is a primary tauopathy characterized by tau protein accumulation. Abnormally phosphorylated tau protein deposits in both neurons and glia with a predilection for the pallidum, subthalamic nucleus (STN), red nucleus, substantia nigra, pontine tegmentum, striatum, oculomotor nucleus, medulla, and dentate nucleus.^12^, ^13^, ^14^, ^15^ This distribution contributes directly to the risk of falls through the indirect locomotor pathway and impairment of the pedunculopontine nucleus (PPN). Notably, this is in contrast to the direct locomotor pathway between the motor cortex and spinal cord, which appears relatively spared in those with parkinsonian gait dysfunction.^16^

**Figure 1:**
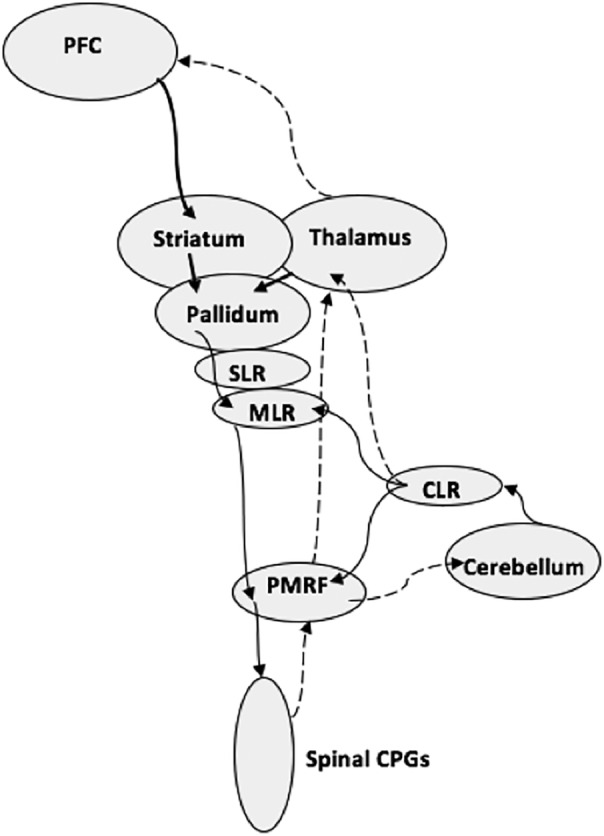
The indirect locomotor pathway. Solid arrows: efferent arm of feedback loop. Dashed arrow: afferent arm of feedback loop. Figure adapted from ref. 16. CLR, Cerebellar locomotor region; CPG, central pattern generator; MLR, mesencephalic locomotor region; PMRF, pontomedullary reticular formation; PFC, prefrontal cortex; SLR, subthalamic locomotor region.

The indirect locomotor pathway is involved in modulating ambulatory movements such as turning.^8^ It comprises connections between the prefrontal cortex, STN, and the pedunculopontine/cuneiform nucleus complex, which are thought to regulate locomotion (Fig. 1).^8^, ^16^ In an Fluorodeoxyglucose‐(FDG)‐Positron Emission Tomography (PET) imaging study of patients with PSP, Zwergal and colleagues^17^ identified dysfunction in the indirect locomotor pathway including in the prefrontal gyrus and thalamus. In this study, clinical measures of gait impairment were inversely proportional to regional cerebral glucose metabolism in the thalamus and STN in patients with PSP.^17^ This is intriguing given the confirmed pathological predilection for the STN in PSP and the well‐known role of the prefrontal cortex and thalamus in regulating STN function via the basal ganglia motor loops or potentially via the ventro‐lateral prefrontal cortex–STN hyper‐direct pathway.^4^, ^18^ Furthermore, Bluett and colleagues^8^ compared the clinical features of a group of people with PSP stratified into frequent and infrequent fallers. Frequent fallers were noted to have worse clinical scores of turning. The authors defined turning as a form of modulated ambulation attributable to the indirect locomotor pathway. This clinical, pathological, and radiological evidence supports a prominent role of the indirect locomotor pathway in the pathophysiology of falls in PSP.

The PPN is another putative neural substrate of falls in PSP; it is situated in the caudal midbrain and rostral pontine tegmentum and densely connected to the basal ganglia and several other networks. Its activity is modulated during locomotion, and it is thought to be a major contributor to motor control. PPN degeneration is seen in postmortem human brains in Parkinson's disease (PD) and other parkinsonian disorders, including PSP.^19^ Dugger and colleagues^20^ showed high tau burden in the PPN of PSP patients, suggesting that this brainstem nucleus may have a critical role in mediating the falls in PSP. The PPN has also been shown to exhibit reduced levels of choline acetyltransferase in PSP, further implicating it in the pathology of the disease.^21^ A brief report of a patient with bilateral PPN infarcts and subsequent gait freezing further supports the role of the PPN in locomotion and potential contribution to falls in movement disorders.^22^ As such, there is clinical and radiological evidence linking gait dysfunction in PSP to the PPN.

### The Natural History of Falls in PSP

Falls within the first year of onset constituted a core criterion in the 1996 National Institute of Neurological Disorders and Stroke and the Society for PSP (NINDS‐SPSP) clinical research criteria for diagnosis of PSP.^14^, ^23^ This was corroborated by several small studies: Nath and colleagues^24^ established that mobility issues were the most common symptom at disease onset, whereas Macia and colleagues^25^ identified that unexplained falls invariably occurred in the first year after a diagnosis of PSP. This restriction to 1 year was relaxed by Bensimon and colleagues^26^ to 3 years to maximize sensitivity for clinical trial recruitment. Later diagnostic criteria adopted this change.^27^ These new criteria were later compared with NINDS‐SPSP, showing a higher sensitivity overall.^28^

Goetz and colleagues^29^ showed progression of gait impairment in the early years of PSP using a composite measure of gait disturbance combining loss of independent walking, inability to stand unassisted, and wheelchair use. People with PSP met this composite milestone of gait disturbance at a median of 57 months from symptom onset. Litvan and colleagues^30^ explored the natural history of PSP in 24 patients. Falls were seen in the first year after diagnosis in 58% of patients, whereas after a mean of 3.7 years after disease onset, 83% of patients reported falls. Two years later, all of the patients reported falls.^30^ Given the retrospective nature of this study and the lack of specification of timing between the last clinical encounter and death, it may not have completely captured the natural history in the latter stage of the disease.^30^ In most of the patients identified by Golbe and Ohman‐Strickland's 2007 study, falling was the first symptom of PSP.^31^ Although this was a prospective study, engagement was more difficult in the later stages of the disease because of the increasing immobility and resulting difficulty traveling to clinic appointments.

A limitation of the aforementioned studies is the lack of pathological confirmation of PSP in the majority of patients. This issue was addressed in a retrospective study by O'Sullivan and colleagues.^32^ Frequent falls were identified in 82% of patients, and the average number of years from disease onset to identification of frequent falls was 3.9 ± 2.5 years. In O'Sullivan and colleagues’ cohort, frequent falls were the most common first clinical milestone reached by patients with PSP (63.6%).^32^ For the purpose of this study, frequent falling was defined as “falls occurring more than twice per year, or the documentation of ‘frequent’ or ‘regular’ falls.” This arbitrary definition that partly relies on the recording in medical notes might not delineate the nature of falls throughout the disease course with accuracy and could be prone to inaccurate reporting. In a study that included pathological confirmation in a subset of patients (11 of 25 who died), Arena and colleagues^33^ prospectively assessed 35 patients with PSP, of whom 34 presented with falls before their first assessment. Patients had fallen a median of 20 times in the previous 12 months.

Falls risk increases with disease progression, but the nature of falls in the later stages of disease is unclear in part because of patient and carer adaptation to multifactorial mobility problems. Although wheelchair use might be expected to reduce falls, one can fall from a chair and certainly during transfers to and from the chair. In addition, the impulsivity of PSP may lead wheelchair users to stand and fall if unattended. As such, the latter part of the natural history of falls in advancing disease course remains unclear.

**Figure 2:**
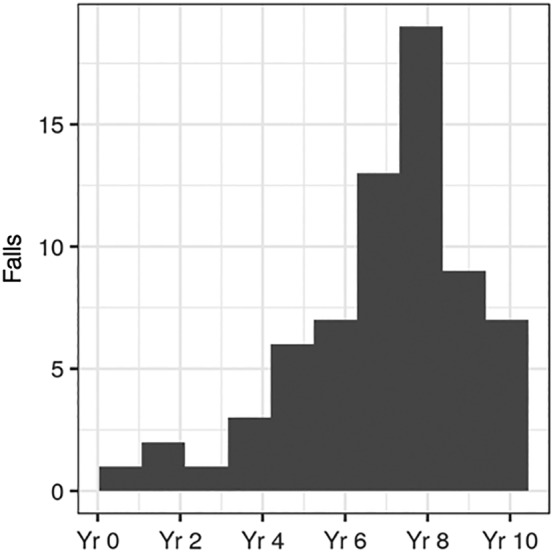
Falls in a single individual with progressive supranuclear with Richardson's syndrome recorded prospectively by their main caregiver, demonstrating a gradual increase during the first 8 years and reduced falls in advanced disease stages. Yr, year.

We report the experience of a single person with PSP‐RS whose main carer diligently and prospectively recorded the number of falls throughout the entirety of their illness and gave postmortem assent to use the data (Fig. 2). Although from a single patient, the detailed record‐keeping matches our clinical impression that the frequency of falls first increases early in the disease and is reduced in later stages.

Does the number of falls usually decrease near the end of the disease as observed in our single patient? A challenge is that falls prior to diagnosis may go unrecorded and unrecognized as a feature of a disease such as PSP, clouding our understanding of very early clinical manifestations of the condition. Furthermore, falls with injury will likely create a recall bias, whereas falls back onto a bed or chair may be more likely to go unrecognized than falls to the floor. “Near falls,” in which a carer reactively and successfully supports an unbalanced patient, may also go unrecorded.

### Measuring Falls and Balance

Measuring how often people fall and the consequences of falling is critical to assessing the burden they impose on the patient, their carer, and wider society and to monitoring response to treatment. Currently, clinical practice and many studies rely on simple rating scales. A number of scales have been established for use in PD such as the Unified Parkinson's Disease Rating scale (UPDRS).^34^ Many studies investigating falls in PSP have co‐opted this scale. It consists of a series of questions with graded answers that help rate the severity of typical symptoms. Part III of the UPDRS or UPDRS–Movement Disorder Society includes clinical assessment of postural stability, which may be interpreted as a falls risk, but there is no direct quantification of actual falls. Application of the UPDRS–Movement Disorder Society in PSP may be limited by cognitive impairment, reducing reporting accuracy. A lack of focus on falls in the UPDRS–Movement Disorder Society means it provides an incomplete description of PSP. Specific to PSP, the PSP Rating Scale (PSPRS) was published in 2007 and incorporates the frequency of falls.^34^ It provides 5 possible responses (0 to 4, with 0 being normal and 4 being wheelchair bound or having more than 30 falls per month) to a single item that quantify the number of falls patients have suffered in the past month. Some of the ranges provided are broad and as such someone who fell 5 times in 1 month would be given a score of 3, as would someone falling 30 times per month. These delineations therefore provide only crude monitoring of the frequency of falls. Furthermore, rating scales reliant on patient or carer responses are inherently subjective and what constitutes a fall is not operationalized. These approaches are further hampered by not controlling for patients acquiring walking aids that may reduce falls and the impact of PSP‐related symptomology on falls such as apathy, disinhibition, and downgaze palsy. Technological solutions may assist in providing a more objective and real‐time assessment of falls. Wearable devices, such as a watch, can provide remote, objective evidence of falls from the patient's home^35^ but have not yet been applied to PSP. Measuring balance may help to identify those with PSP at risk of falling. One approach successfully applied to PSP is to use a modified turning test that evaluates a patient's ability to turn 180° from a standing position.^8^

Could the methods used in other conditions be useful in PSP, such as gait laboratory analysis?^36^, ^37^ Using video cameras and reflective markers, Sofuwa and colleagues^36^ measured kinetic and kinematic variables in patients with PD when compared with healthy controls. This approach detected significant differences between kinetic profiles in the 2 groups, providing an objective, quantitative measure of balance. Although objective, these approaches require specialist staff and equipment and may be subject to observer bias and artificially elevated performance as a result of the increased attention. Measuring gait formally is largely restricted to laboratory environments.^38^ Body‐worn monitors are an emerging method to monitor gait with a similar ability to detect gait abnormality in PD.^38^ These ambulatory devices can be given to patients and data collected remotely, allowing longer evaluation periods and ameliorating some of the drawbacks of the laboratory setting. Indeed, these wearable devices have been shown to be able to identify patients at increased risk of falling in PD.^39^

Current clinical and research practice in measuring falls is guided by questionnaires such as the PSPRS. Measuring falls clearly presents a challenge, further compounding the difficulty of research in this area. A uniform, reliable way of measuring falls would improve attempts to measure response of falls as a clinical parameter to treatment in PSP. The gold standard would be prospectively recording a falls diary, although this may not be practical for day‐to‐day use. Better validation of rating scales and balance tests is required before they can reliably be said to predict the rate of falls. Emerging technological solutions may provide the best prospective and objective assessment of falls and balance.

### Fall Prevention

Given the risk of fractures and the economic and societal cost, minimizing falls and their consequences in PSP should be a priority. Here we explore the medical and nonmedical approaches to preventing falls and the associated morbidity.

#### Medical Therapies

Despite trials of various agents, there are currently no effective pharmacological options to prevent disease progression or reduce falls in PSP.^40^ Levodopa and dopamine agonist therapy, although mainstays in PD, have limited effect in PSP, and an effect on falls has not been demonstrated.^41^, ^42^

Given the paucity of evidence in PSP, can we learn anything from attempts to prevent falling in PD? Cholinesterase inhibitors may reduce the number of falls in PD: a randomized, double‐blind, placebo‐controlled study compared rivastigmine with placebo in PD and found a 45% reduction in falls in the treatment group.^43^ There is similar evidence that donepezil and galantamine reduce fall frequency in PD.^44^, ^45^ Potential anticholinesterase responsiveness corroborates the suggestion that acetylcholine deficiency may be involved in PSP pathophysiology, as discussed previously.

Agents exploiting improved understanding of the pathophysiology of PSP have been investigated.^40^ Only coenzyme Q10 has been shown in a double‐blind, randomized, placebo‐controlled trial to have a modest clinical effect, but this was not replicated in a further, larger trial.^46^, ^47^ Notably, this trial showed improvement in the overall PSP rating scale and gait scale; the specific effect on falls and freezing was not reported. Although based on hypotheses derived from putative disease mechanisms, other agents have not shown any clinical benefits, including for falls.^26^, ^48^, ^49^

In summary, no evidence‐based therapeutic options exist for preventing falls in PSP. A review of http://clinicaltrials.gov shows there are 3 active trials investigating medical treatment in PSP with falls or PSPRS as primary outcome. One, Efficacy of RIVAstigmine on Motor, Cognitive and Behavioural Impairment in Progressive Supranuclear Palsy (RIVA‐PSP), is investigating rivastigmine with falls as the primary outcome. Evidence from PD suggests that agents such as acetylcholinesterase inhibitors may be beneficial for falls, but as yet this has not been sufficiently assessed in PSP.

#### Nonmedical Approaches

Only tentative evidence exists for preventing falls in patients with PSP with nonmedical approaches. Here we examine this limited evidence but also include relevant studies of fall prevention in PD. Although distinct clinical entities, this may inform future research directions in PSP.

Exercise Training and Physical Therapy. Physical therapy is used widely in PSP, although there is limited evidence to support which interventions are effective. Clerici and colleagues^50^ compared standard treadmill training with robot‐assisted therapy in 24 people with PSP. Although there was little difference between the 2 interventions, both reduced the number of falls and improved scores on the PSP‐RS and Berg Balance Scale. Limitations of this study are the lack of a control group and uncertainty regarding how long the improvement in propensity to fall would continue: the study program lasted only 4 weeks.^50^ The use of a treadmill‐based physical therapy program is supported by the case study of a single patient with PSP whose falls reduced after an 8‐week program.^51^ Although this evidence is not definitive, it demonstrates the feasibility of treadmill training in PSP.

Two small studies have used biofeedback methods to improve balance in PSP. In one study, auditory biofeedback in 8 patients with PSP demonstrated improved posture and dynamic balance that was sustained at 4 weeks.^52^ This study employed instructions delivered by headphones attached to a wearable device, although there was no control group. In a second study, visual biofeedback in addition to balance training improved outcomes against balance training alone.^53^ This study assessed 19 people with PSP and included a task involving changing the direction of gaze with auditory feedback. Although small, these studies demonstrated that biofeedback approaches are feasible in PSP.

Interventions to reduce falls in PD have been explored. Ashburn and colleagues^54^ compared a 6‐week community physiotherapy program with normal care; 9% fewer people fell in the intervention group, although this result was statistically insignificant.^54^ The rates of near falls and repeated near falls were significantly reduced in the intervention group, however. Goodwin and colleagues^55^ compared an exercise program versus usual care. After a 10‐week physiotherapy program with further home exercises, 4% fewer people had fallen in the intervention group. This difference was not statistically significant.^55^

Both treadmill training and biofeedback approaches to improve balance in PSP have shown signs of promise in very small studies and warrant further investigation.^50^, ^52^ Studies in PD may help to guide the future direction of nonmedical interventions for PSP, although despite some similarities, they are distinct clinical entities. The application of these data to PSP should be cautious given the differences in clinical presentation and frequency of falls between PSP and PD. It is possible that PSP subtypes with clinical presentations more similar to PD, such as PSP‐parkinsonism, may be more amenable to treatments with proven effects in PD.

Deep Brain Stimulation (DBS). Given the role ascribed to the PPN in locomotion and its apparent degeneration in PSP (and PD), some studies have explored DBS of this area to reduce fall frequency in these diseases. A study on unilateral DBS of the PPN in 6 patients with advanced PD showed that the treated patients reported a significantly reduced number of falls after 1 year.^56^ However, other studies of PPN DBS in PD have reported mixed results.^57^

Applying methods similar to those employed using DBS in 3 patients with PSP, Servello and colleagues^58^ showed an improvement in falls and balance, as reported by patients, and an average PSPRS score reduction of 26% after 12 months. Another 2 small trials of unilateral PPN DBS have shown modest benefit overall but no specific effect in the domain of falls.^59^, ^60^ A study of bilateral PPN DBS in PSP failed to replicate this reduction in falls, but did show improvement in some gait parameters and a reduction in hypokinesia.^60^

The studies exploring PPN DBS in PSP thus far have all been conducted in small samples and without control groups. The mixed results of these studies may reflect the diffuse and interconnected nature of the neuroanatomical substrate of locomotion, the degeneration of which underpins falls, and of which the PPN is only 1 component.

Even if proven to be effective, it is likely that only limited numbers of patients with PSP would be eligible for DBS insertion for reasons of cognitive, psychiatric, or other medical comorbidities.^61^

### Reducing Risk

In addition to interventions to try and prevent falls, minimizing the risk factors of falling might be another important mechanism to reduce the fall‐related morbidity and associated medical costs.

Polypharmacy is one such risk factor and is strongly associated with falls in the elderly. One U.K. study has shown that using 5 or more drugs was associated with a 21% increased rate of falls during a 2‐year period in those aged older than 60.^62^ Specific drugs carry a particularly high risk of falls, including drugs for symptoms often encountered in PSP. Nocturia in PSP relates to bladder instability and can also contribute to falls, especially if patients are trying to reach the bathroom or commode at night in low light, unattended, and without time to adjust to postural and thermal shifts on getting out of bed. Anticholinergic drugs are often prescribed for urinary symptoms but are a risk factor for falls in older people.^63^, ^64^ Noncholinergic agents such as mirabegron offer potential advantages for bladder instability, urgency, and nocturia in PSP while noting the lack of evidence from direct trials.

Other medication commonly prescribed in the elderly population, including people with PSP, include Selective Serotonin Reuptake Inhibitors (SSRIs), opiates, tricyclic antidepressants, and benzodiazepines. SSRIs are effective for the emotional lability of PSP (especially the pseudobulbar affect), even in the absence of depression, but this class of drugs is a risk factor for falls in the general population.^65^ Opiates for pain, ironically used after falls, may exacerbate the risk of fall recurrence, whereas the cholinergic side effects of tricyclic antidepressants are a significant potential hazard.^64^ Benzodiazepines, used for sleep, anxiety, or agitation, can further increase the risk of falls in patients with diverse illnesses,^66^ and there is no reason to believe that this risk does not extend to those with PSP.

Falls in PSP are often precipitated by impulsivity and a tendency to stand and walk despite the known risks. This is sometimes referred to as the “rocket sign” of PSP or motor recklessness. We have elsewhere reviewed the approach to cognitive changes in PSP, including impulsivity, and recommended an individualized therapy as the best approach to managing impulsive behavior in PSP, including carer support, education, and environmental consideration supporting drug‐based approaches.^67^ In the frontal variant of PSP, citalopram and trazodone are options to consider where there are significant impulsive behaviors based on class 2 or below evidence from the related condition of frontotemporal dementia.^68^, ^69^

Given the inevitability of falls and high risk of a fracture, it is appropriate to minimize the risk of fracture by addressing bone density. The Fracture Risk Assessment Tool (FRAX) score is a well‐validated tool to assess fracture risk in the general population and has normalized data for specific countries (https://www.sheffield.ac.uk/FRAX/tool.aspx). In the United Kingdom, it is often used in conjunction with the National Osteoporosis Guideline Group's intervention threshold to guide the appropriate time to start treatment.^70^

The National Osteoporosis Foundation recommendations for pharmacologic treatment of osteoporosis are based in part on the World Health Organization 10‐year fracture probability model. These recommendations are based on cost‐effectiveness and should be used together with other considerations when making treatment decisions for individual patients. Among the other considerations is the exceptionally high risk of falls in PSP and 25% lifetime incidence of fracture.^10^ We therefore recommend bone densitometry in all patients with PSP and proactive management of osteopenia and osteoporosis.

The most commonly prescribed treatments for osteoporosis are bisphosphonates. In the United Kingdom, weekly oral alendronic acid is common. However, bisphosphonates require special steps to prevent oesophagitis and oesophageal ulcers, including upright posture and a high volume of water to follow the tablet: these safety measures are often not practical with the dysphagia and neck dystonia of PSP. It may therefore be necessary to consider alternatives such as denosumab (a fully humanised monoclonal antibody against receptor activator of nuclear factor kappa B ligand), teriparatide (recombinant human para‐pthyroid hormone), or raloxifene (an oestrogen receptor modulator).

Another important risk following falls is intracranial hemorrhage. A challenging area for the clinical management of falls in PSP is whether to continue antiplatelet or anticoagulant therapy in a person with frequent falls given the risk of intracranial hemorrhage balanced against the thrombotic risk of not being on these medications. Anticoagulation is underprescribed for the elderly, and it has been suggested that the risk of falls in the elderly population has been overstated as a reason to discontinue or not initiate anticoagulation.^71^ However, most studies of anticoagulation exclude people who fall regularly, and in those that do include patients with falls, the frequency of falls is likely to be an order of magnitude lower than in people with PSP. There is no trial evidence to help guide decision making, and we advise assessment of an individual level of risk and a discussion of the dilemma with patients and carers. For those on warfarin, an unstable dosage with intermittently excessively high INR presents a particular risk, and the direct oral anticoagulants as single‐dose alternatives may be considered, particularly because options for reversing the anticoagulation effect of direct oral anticoagulants are emerging.^72^ Although data are lacking in PSP cohorts, there are large studies of the risk of traumatic intracranial hemorrhage in those on anticoagulants or antiplatelet agents.^73^, ^74^, ^75^ Approximately 7% to 15% of those admitted to emergency care following falls, including ground‐level falls among elderly patients, were associated with radiological evidence of intracranial hemorrhage. Perhaps surprisingly, anticoagulation did not significantly increase the risk. These data pertain to falls with admission to emergency care, not all community falls. For those with definite indications for anticoagulation, and only occasional falls, the current evidence would seem to favor remaining on treatment, but where falls are frequent, the case should be reviewed with input from hematology and cardiology teams where necessary.

### To Fall or Not to Fall?

Given the limited efficacy of current prevention strategies, falls remain an inevitable part of PSP. Therefore the person with PSP, their family and carers, and clinicians must make choices on weighing the risk of falls against the benefits of continuing to mobilize. There is no evidence we are aware of to inform this decision in PSP. However, the benefits of physical exercise in later life are well documented on balance,^76^, ^77^ the prevention of osteoporosis,^78^ and well‐being.^79^ This benefit extends to frail elderly populations where physical activity can prevent a loss of functional autonomy (odds ratio 0.67).^80^

On the other hand, falls are not benign events. In addition to the physical consequences, falls in older adults are associated with significant anxiety^81^, ^82^ and a reduced quality of life.^83^ In some people, this anxiety extends to symptoms of posttraumatic stress disorder.^84^

In general, our practice is to advocate for maintaining an active and independent life as far as possible while recognizing the risk of falls. This is encapsulated in the concept of positive risk‐taking, which has been promoted for mental health conditions.^85^ This approach looks to promote function and independence while promoting a no‐blame culture when things go wrong. Relevant for medical and paramedical professionals and those working in institutional care, Morgan^85^ suggests that this approach needs to be supported by local leadership given the natural tendency to avoid risk: “It should be the explicit role of senior management to understand and clearly articulate the rationale for positive risk‐taking, to instill the necessary confidence in staff to take carefully considered risks in pursuit of beneficial outcomes.” Ultimately, each person with PSP needs to be involved in a personalized approach and the discussion of the benefits and risks of staying active.

## Conclusion

Falls are at the core of the diagnosis and presentation of PSP, and they are associated with significant morbidity and cost to the health system. Pathological and neuroimaging evidence implicate the PPN and indirect locomotor dysfunction in the pathophysiology of falling PSP. Although the centrality of falling is well established in PSP, it is unclear how the frequency of falls change throughout the disease course, particularly later in disease.

Measuring falls is largely dependent on retrospective assessment and rating scales that carry inherent biases and are not necessarily designed with falls in mind. This compounds the difficulty of studying this symptom and any treatment effects. Although prospective counting of how many times a person falls to the floor would provide the best quantitative data for future studies of falls, the assessment of balance and turning may provide a useful approximation of falls risk.

Attempts to prevent falls by medical and nonmedical means are not yet established. Physical therapy approaches, treadmill exercises, and biofeedback methods may be beneficial, but randomized controlled trials in PSP patients are lacking. There are no proven pharmacological options for preventing falls in PSP, although addressing the risk factors for falls such as polypharmacy, nocturia, and impulsivity may be beneficial. Among candidate medical therapies, cholinesterase inhibitors show promise in PD and pure gait freezing but are as yet unproven in PSP. The comorbidity associated with falls may be reduced by addressing osteoporosis and fracture risk and assessing the need for anticoagulation therapy when appropriate. Each person with PSP requires a personalized discussion about maintaining mobility and the risk of falls for which we advocate a positive risk‐taking approach.

## Author Roles

(1) Manuscript Preparation: A. Writing of the First Draft, B. Review and Critique.

F.S.B.: 1A, 1B

J.B.R.: 1B

L.P.: 1B

T.R.: 1A, 1B

## Disclosures

**Ethical Compliance Statement:** The authors confirm that the approval of an institutional review board was not required for this work as this is a review article. Verbal consent for the use of anonymized patient information in Figure 2 was obtained from the patient's spouse after their death. We confirm that we have read the Journal's position on issues involved in ethical publication and affirm that this work is consistent with those guidelines.

**Funding Sources and Conflict of Interest:** No specific funding was obtained for this work. The authors declare no conflict of interest.

**Financial Disclosures for the Previous 12 Months:** F.S.B. is employed by Cambridge University Hospital NHS Trust. J.B.R. is supported by the Wellcome Trust (103838), the National Institute for Health Research Cambridge Biomedical Research Centre, and the Cambridge Centre for Parkinson‐plus. J.B.R. has received consultancy fees from Biogen, Asceneuron, Lilly, and Astex; research grants from Janssen, AZ‐Medimmune, Lilly, the McDonnell Foundation, Medical Research Council, PSP Association, Parkinson UK, Alzheimer Research UK, and Evelyn Trust; and he serves as Associate Editor for *Brain*. L.P. is supported by MRC Research Grant MR/P01271X/1. T.R. is employed by the Cambridge Centre for Parkinson's Plus (University of Cambridge), the Brain Repair Centre (University of Cambridge), Cambridge University Hospital NHS Trust, and West Suffolk Hospital NHS Trust. T.R. has received honoraria from National Institute for Health and Clinical Excellence, Oxford Biomedica, and Learna Ltd.
